# Acute irreducible volar distal radioulnar joint dislocation treated with open reduction through dual approaches: A case report and literature review

**DOI:** 10.1016/j.heliyon.2022.e11222

**Published:** 2022-10-25

**Authors:** Osama Z. Alzobi, Mazhar Fuad, Ayman Abdolenour, Ashraf T. Hantouly, Hammam Kayali, Fadi Bouri

**Affiliations:** Department of Orthopaedic Surgery, Hamad Medical Corporation, Doha, Qatar

**Keywords:** Distal radioulnar joint dislocation

## Abstract

**Introduction:**

Acute dislocation of distal radioulnar joint (DRUJ) is a rare pathology. Most cases were managed with closed reduction, and few patients required open reduction through dorsal or volar approaches. We describe a patient who required open reduction using dual approaches.

**Case presentation:**

This paper reported a case of acute DRUJ volar dislocation that failed closed reduction. Open reduction was tried using a dorsal approach which failed to achieve joint reduction. A second volar approach to release volar joint capsule had only achieved joint reduction.

**Discussion and conclusion:**

This case report highlighted the importance of open reduction when treating acute DRUJ injuries that failed closed reduction. We strongly recommend that surgeons should be ready to utilize dual approaches for this injury and to appraise patients about this possibility.

## 1 Introduction

Acute isolated volar distal radioulnar joint (DRUJ) dislocation is a rare injury that is usually misdiagnosed and treated inappropriately, especially in a busy trauma center [1, 2]. DRUJ has complex anatomy with inherent instability in the relation between distal radius and ulna; therefore, any injury to this joint will affect forearm axis of rotation with direct effects on protonation and supination [3].

Volar DRUJ dislocations are less frequent than dorsal ones; therefore, very few reports in the literature have described volar dislocations [4]. Most studies have reported on volar DRUJ dislocation were managed with closed reduction. Failure of closed reduction mandated an open approach. However, there is a paucity of literature with regard to open reduction treatment for those who failed closed reduction [2, 3, 4, 5]. In all case reports in the literature highlighting open reduction treatment, only one approach was always required and adequate to address all blocking structures [2, 3, 4, 5, 6, 7, 8, 9, 10]. The novelty of our report is that a second approach was not needed for reduction in any of the previous case reports, and was used in our case. Therefore, we reported a case of isolated irreducible volar dislocation of DRUJ, that failed closed reduction, failed open reduction using a dorsal approach alone, and was managed with open reduction using dual approaches. A thorough review of the literature on such cases was done.

## 2 Case presentation

The Surgical Case Report (SCARE) statement guidelines were utilized to report this case.

**Figure 1:**
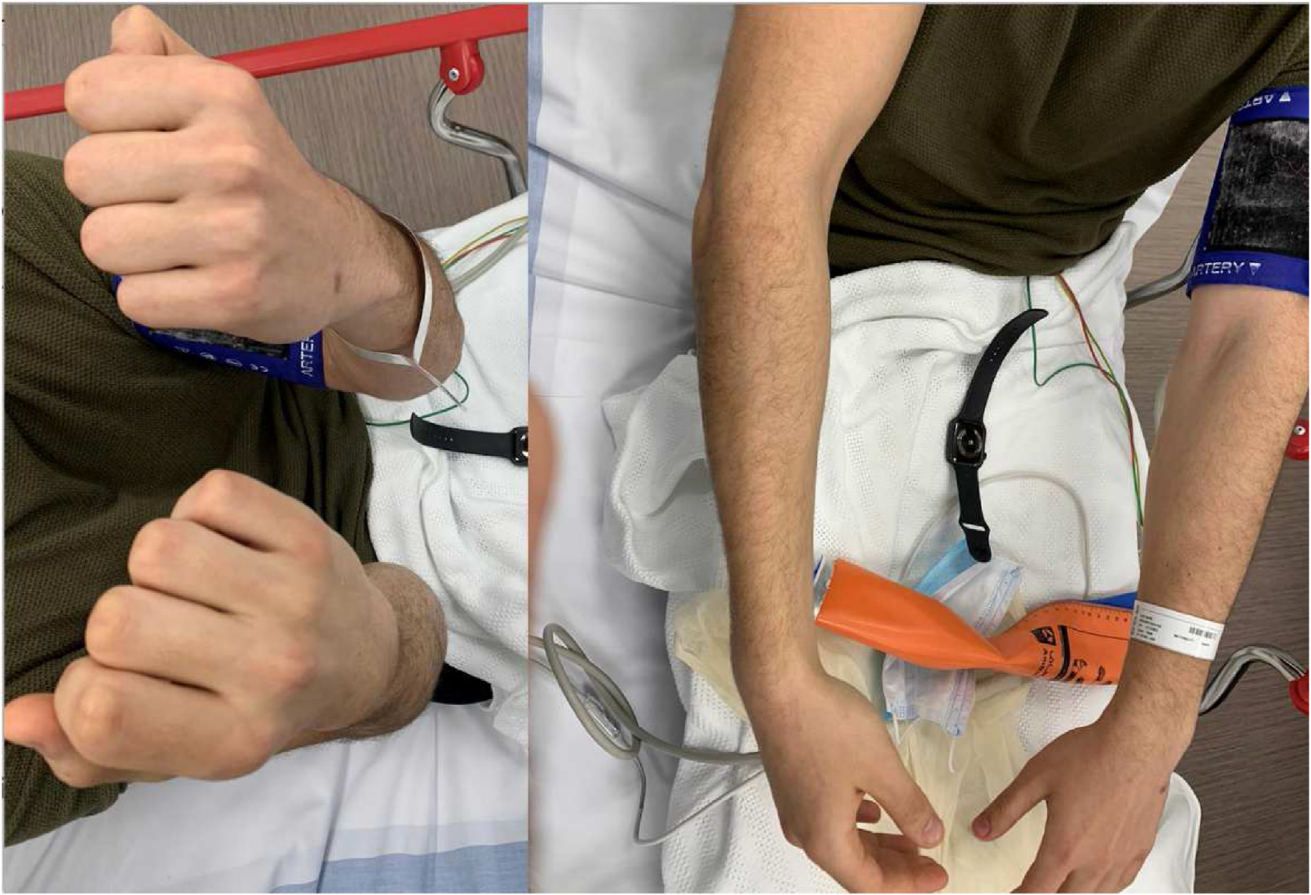
Clinical photos of the patient on presentation.

**Figure 2:**
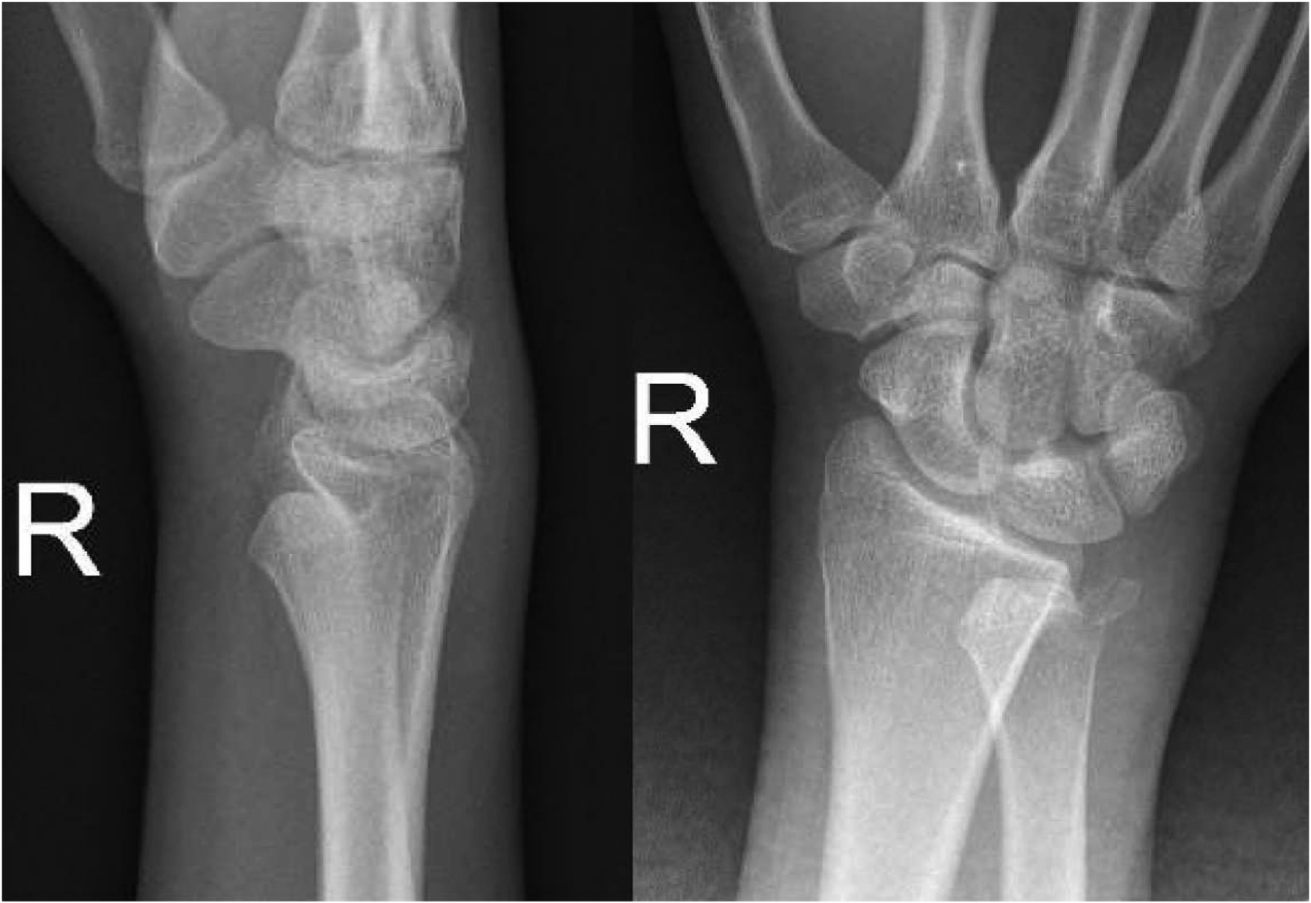
Radiographs of the wrist at initial presentation.

**Figure 3:**
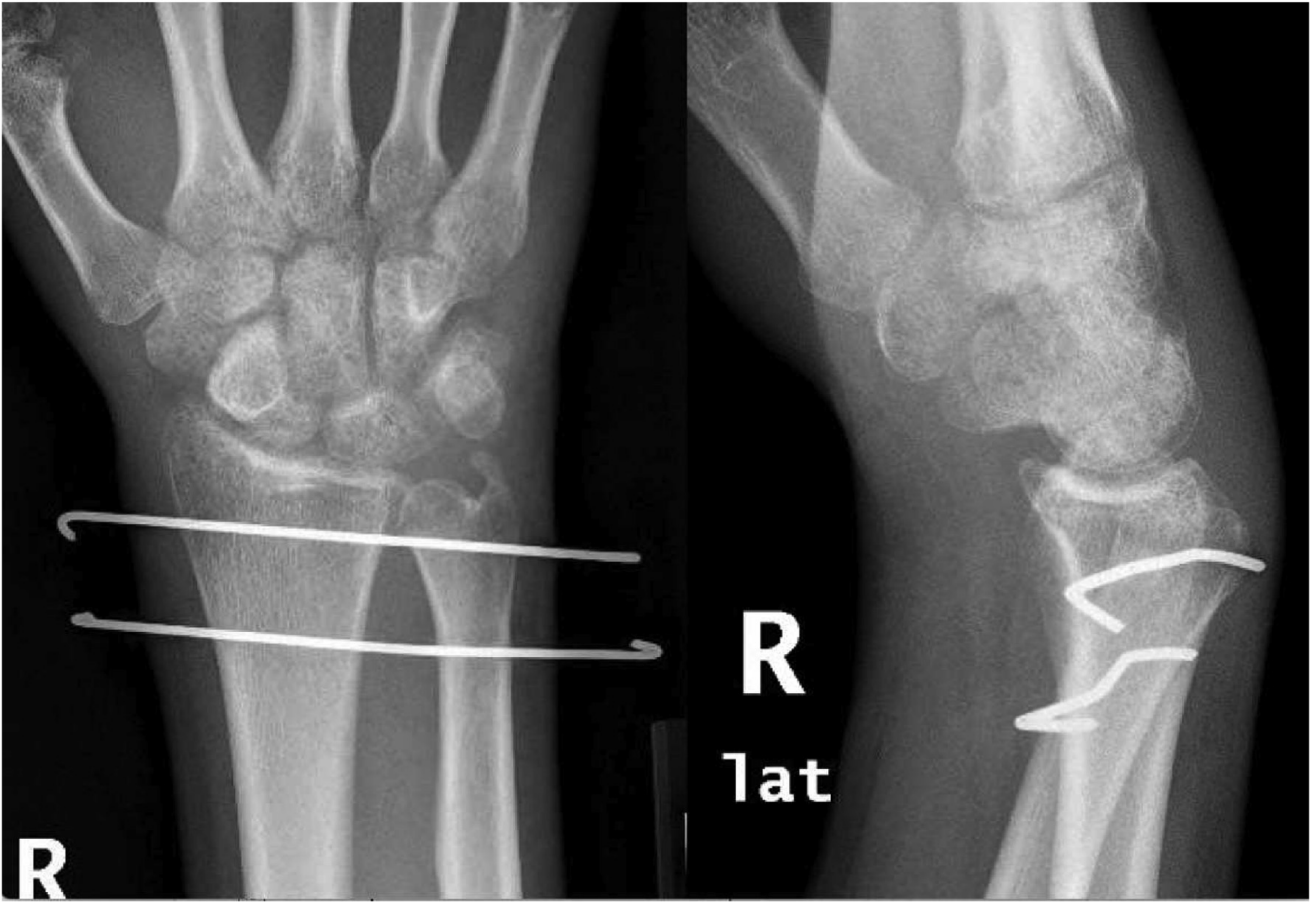
Radiographs of the wrist after reduction.

A nineteen-year-old male, right-hand dominant, presented with right wrist pain and lack of forearm rotation after punching a wall with his fist. He had no previous trauma or medical or surgical history related to that wrist. The patient had no history of smoking or alcohol consumption and no previous corticosteroids intake. On physical examination, his forearm was locked in supination with restricted protonation (Figure 1). The normal prominence of ulna head was absent dorsally and palpable on volar side. Skin and neurovascular status were intact. Initial posteroanterior radiograph demonstrated distal overlap of radius and ulna with associated ulna styloid fracture. A lateral radiograph revealed volar displacement of ulna in correlation to the radius (Figure 2). Closed reduction was attempted under sedation in the emergency department; however, the attempt failed, and the wrist was placed in a temporary splint until the dislocation could be reduced operatively. The patient was then taken to the operating room. A trial of closed reduction under general anesthesia was attempted but also failed. A dorsal approach to DRUJ was utilized over the fifth extensor compartment. The joint was exposed, head of ulna was dislocated volar, and triangular fibrocartilage complex (TFCC) was found intact. Multiple reduction trials were tried using a Freer elevator but failed even after releasing dorsal radioulnar ligament. A second incision on volar side was utilized to approach the joint between Flexor Carpi Ulnaris (FCU) and Flexor Digitorum Superficialis (FDS). Head of ulna was found buttonholing through the capsule and impacted against sigmoid notch of the radius. Reduction was only possible after releasing the volar capsule and disimpacting ulna head. Dorsal radioulnar ligament was repaired; however, the joint was unstable and stabilized with two Kirschner wires across both radius and ulna proximal to DRUJ. The wrist and forearm were placed in a sugar-tong splint in neutral position (Figure 3).

**Figure 4:**
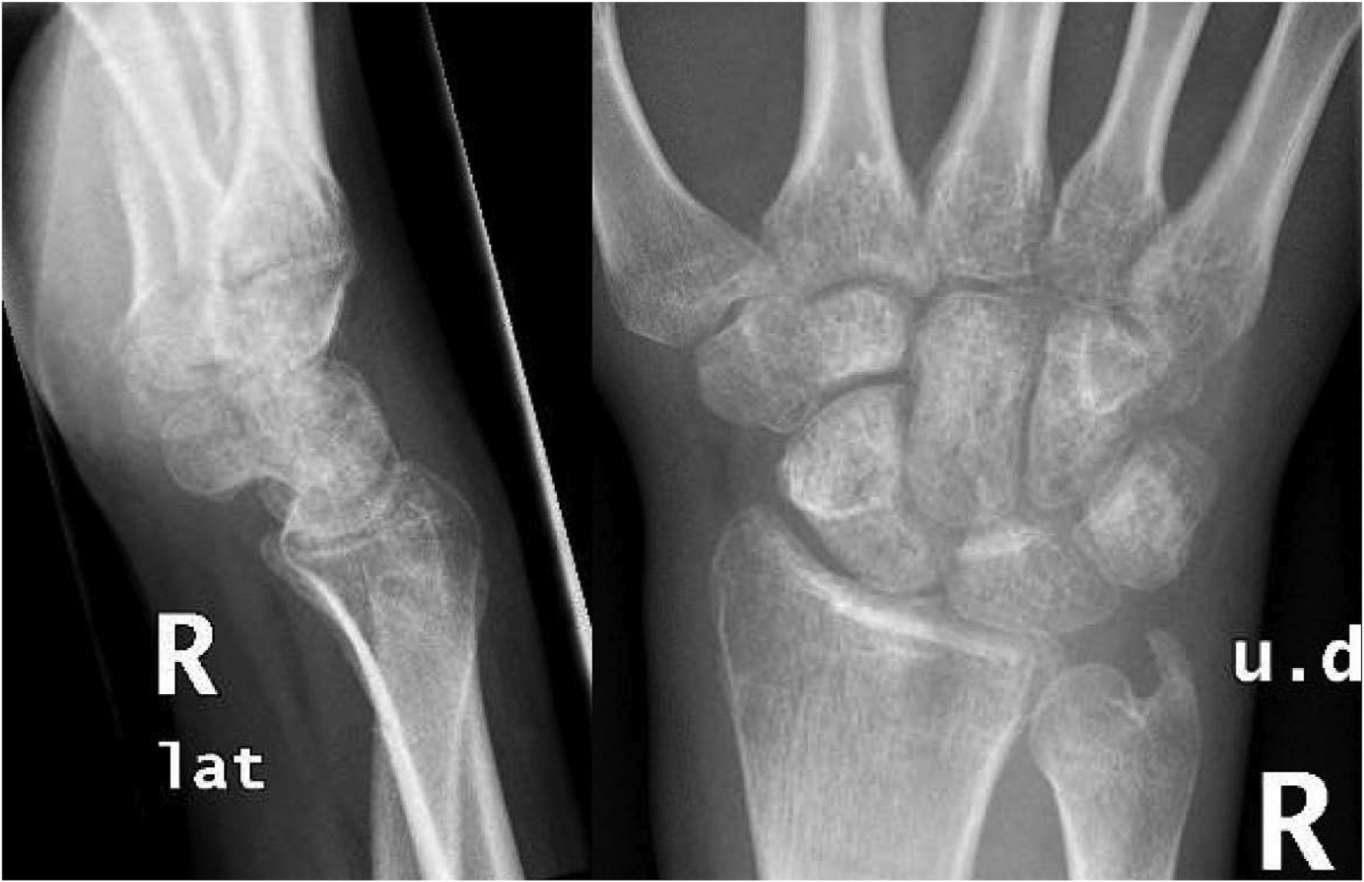
Radiographs of the wrist after Kirschner wires removal.

**Figure 5:**
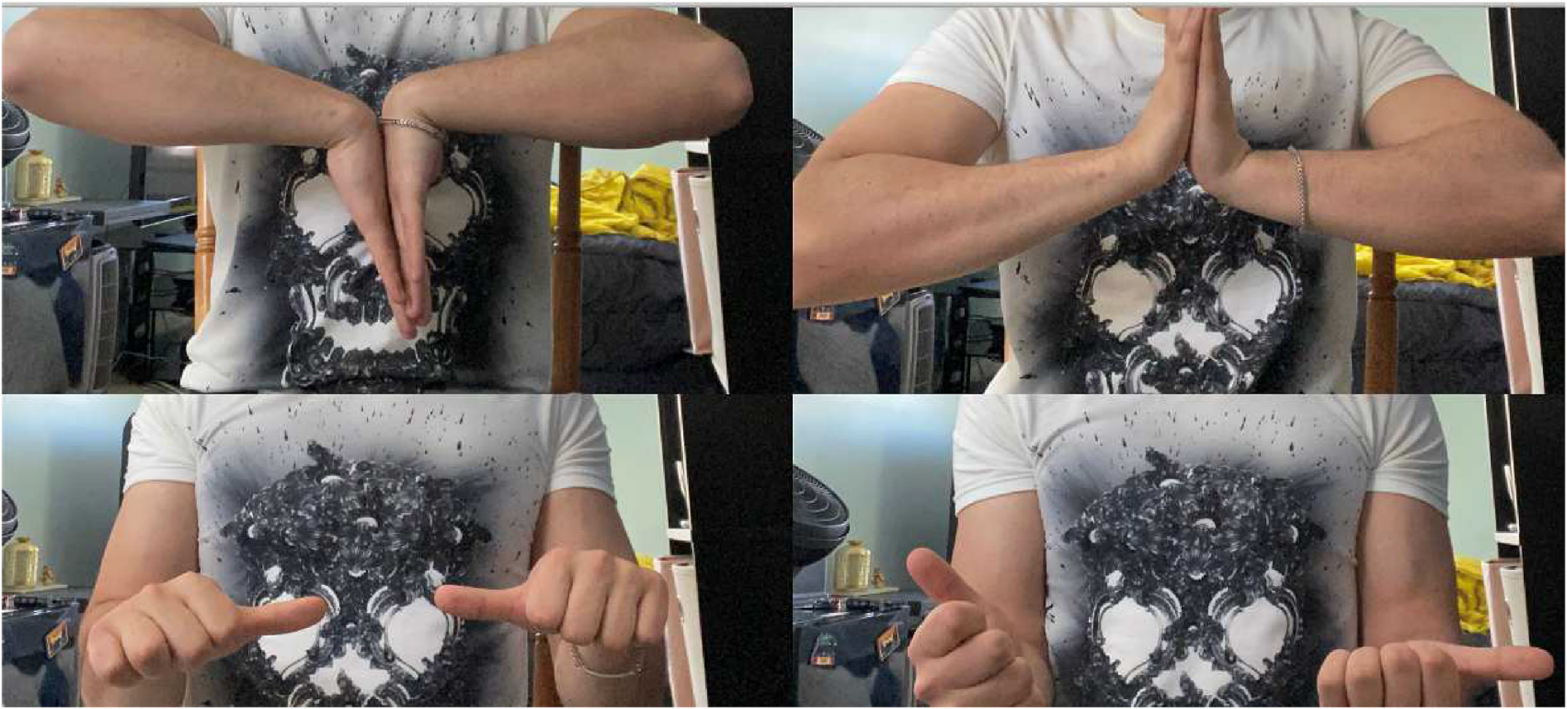
Clinical Photos of the patient after one-year follow-up.

Two weeks later, wound was inspected and pin sites were found dry and clean. Eight weeks after surgery, Kirschner wires were removed, a removable splint was applied, and occupational therapy was started (Figure 4). After one month, the patient noticed significant improvement and was compliant with therapy sessions. His visual analog scale (VAS) score was 0 and Q-DASH score was 22.7. Range of movement was 45/30 for Flexion/Extension arc, 55 degrees of protonation and no supination. Grip strength was 15 kg compared to 42 kg on the other side. Radiographs showed satisfactory alignment of DRUJ. Six months post-surgery, he was satisfied with his wrist motion, participated in all daily activities, and was compliant with therapy sessions. His flexion/extension arc has been improved to 60/50°, protonation/supination arc to 75/40 and Q-DASH score was 18. Range of motion was not changed at one-year follow-up as he could not afford to do physical therapy; however, he was back to regular gym activities. His last Q-DASH score was nine (Figure 5).

### Ethical approval

The case report was approved by the Medical Research Centre at Hamad Medical Corporation, Doha, Qatar. Reference number: MRC-04-22-253.

### Consent

Written informed consent was obtained from the patient for publication of this case report and accompanying images. A copy of the written consent is available for review by the Editor-in-Chief of this journal on request.

## 3 Discussion

The most important finding from this article is that a volar dislocation of DRUJ can fail open reduction through dorsal or volar approaches. Which necessitates the use of a second approach. This article reported a case with acute volar dislocation of DRUJ treated with open reduction through dual approaches. Several described anatomical structures can preclude joint reduction [3]. Block to reduction may include entrapment of extensor carpi ulnaris tendon (ECU), parts of TFCC, impacted fracture of distal part of ulna by rim of sigmoid notch, or even volar joint capsule. In the literature, few reported cases with volar dislocation failed closed reduction; therefore, they were treated with open reduction within the first two weeks (Table 1). In all of these reports, one approach was always required and adequate to address all blocking structures [2, 3, 4, 5, 6, 7, 8, 9, 10]. The novelty of our report is that a second approach was not needed for reduction in any of the previous case reports, and was used in our case.

**Table 1:**
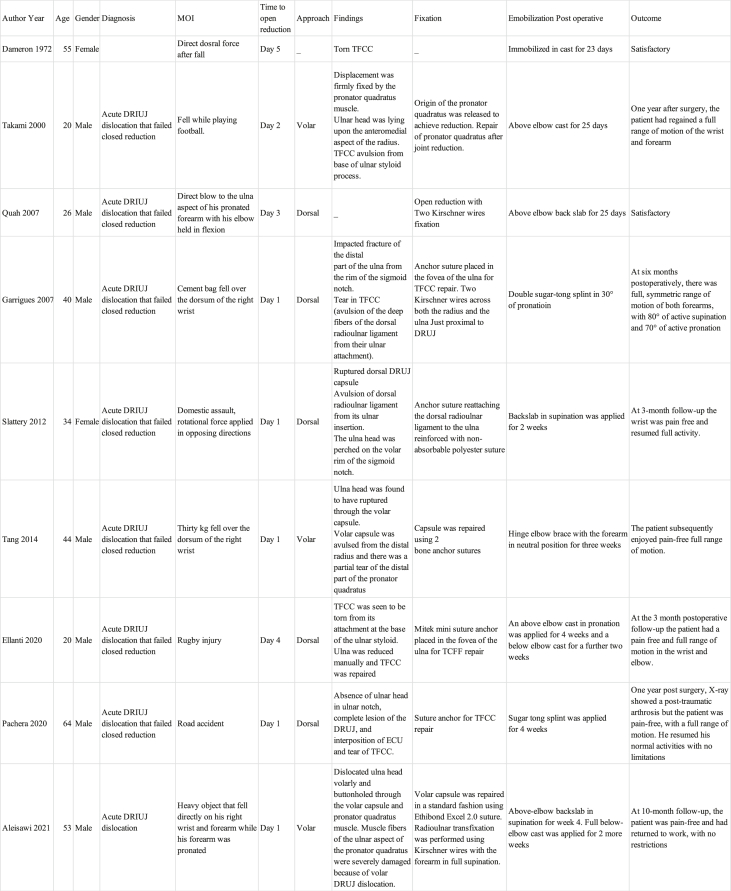
Summary of Acute DRUJ Injuries that Failed Closed Reduction and Treated with Open reduction.

It was first reported in 1972 on a 55-year-old female with a volar DRUJ dislocation and was found to have torn TFCC after open reduction [6]. In 2000, Takami et al. reported another volar DRUJ displacement firmly fixed by pronator quadratus muscle with associated TFCC avulsion. Releasing the origin of pronator quadratus through a volar approach achieved satisfactory reduction [7]. Garrigues et al. [3] and Slattery et al. [8] described perching appearance of DRUJ on a CT scan, where impaction of ulna head on the volar rim of the sigmoid notch resulted in failure of closed reduction. Furthermore, Garrigues et al. described open reduction through a dorsal approach in which TFCC fibers were reattached using an anchor suture, and DRUJ was stabilized with two Kirschner wires [3]. On the other hand, Slattery et al. utilized a dorsal approach and reported a dorsal capsule rupture with dorsal radioulnar ligament avulsion from its ulnar insertion [8]. In this report, DRUJ was stabilized after reduction with anchor suture, reattaching dorsal radioulnar ligament to ulna, which was reinforced with a non-absorbable polyester suture. In Tang et al. [9] report, block to reduction was due to rupture of ulna head through volar DRUJ capsule. Avulsion of volar capsule was reported from distal radius with a partial tear of pronator quadratus. Open reduction was performed using volar approach and the capsule was repaired using two bone anchor sutures [9]. Similarly, Ellanti et al. [5] described open reduction of DRUJ through a dorsal approach. They found a torn TFCC, which was attached after the reduction in fovea of ulna. Pachera et al. [4] reported interposition of ECU and tear of TFCC that was repaired with anchor suture after reduction of DRUJ. Closed reduction was performed initially in all reports, except in the case report by Aleisawi et al. [10], in which they proceeded with open reduction due to comminution of distal ulna found on CT scan. The authors reported volar dislocation of ulna head, which was buttonholed through volar capsule and pronator quadratus muscle. The volar capsule was repaired and DRUJ fixation was performed using Kirschner wires with forearm in full supination [10]. It is important to acknowledge that this review included five articles that reported the use of dorsal approach, three articles studies that reported the use of volar approach, and one article that did not specify the approach. Many surgeons would prefer a dorsal approach for most DRUJ operative procedures [11]. It is important to note that no report in the literature indicated that a volar DRUJ dislocation was irreducible to open reduction using the dorsal approach. In this report, ulna head was found buttonholing through the capsule and impacted against sigmoid notch; therefore, a second volar approach was needed to release the volar capsule and achieve joint reduction.

In conclusion, this case report presents a unique irreducible volar DRUJ dislocation treated with dual approaches, and it highlights the importance of open reduction when treating acute DRUJ injuries that failed closed reduction. We strongly recommend that the surgeon should be ready to utilize dual approaches for this injury and to appraise patients about this possibility.

## Declarations

### Author contribution statement

All authors listed have significantly contributed to the investigation, development and writing of this article.

### Funding statement

The Open Access funding was supported by the Qatar National Library. This research did not receive any specific grant from funding agencies in the public, commercial, or not-for-profit sectors

### Data availability statement

Data will be made available on request.

### Declaration of interest’s statement

The authors declare no conflict of interest.

### Additional information

No additional information is available for this paper.
